# An Integrative Serum Pharmacology-Based Approach to Study the Anti-Tumor Activity of *B. paniculatum* Aqueous Bulb Extract on the Human Hepatocellular Carcinoma Cell Line BEL-7404

**DOI:** 10.3389/fphar.2020.01261

**Published:** 2020-10-02

**Authors:** Xuesong Feng, Guangyuan Ma, Hailong Shi, Yuewen Wang, Xu Chao

**Affiliations:** ^1^ Basic Medical Academy, Shaanxi University of Chinese Medicine, Xianyang, China; ^2^ The Research Department, The Second Affiliated Hospital of Shaanxi University of Chinese Medicine, Xianyang, China

**Keywords:** traditional Chinese medicine, *Bolbostemma paniculatum*, Tu Bei Mu, hepatocellular carcinoma, network pharmacology

## Abstract

The herb *Bolbostemma paniculatum* (Maxim) *Franquet* (Cucurbitaceae family), also known as Tu-Bei-Mu (TBM) in Chinese, has shown curative effects to treat several types of cancer as an adjunctive therapy. Thereby we intend to find its effect on the human hepatocellular carcinoma (HCC) and to understand the pharmacological mechanism behind it. In this study, an integrative serum pharmacology-based approach linking serum pharmacology and bioinformatics prediction was employed. Firstly, we used the serum taken introgastrically from the rats dministered by TBM aqueous bulb extract to culture the HCC cell line BEL-7404 and detect its anti-tumor effects. Secondly, the TBM putative targets were predicted using the ETCM database and known therapeutic targets of NPC were collected from the OMIM database. Then, a TBM-HCC putative targets network was constructed using the DAVID and STRING databases. Thirdly, key gene targets were obtained based on topological analysis and pathway enrichment analysis. The expression of 4 representative key targets were validated by Western blotting. As a result, 36 TBM targets and 26 known therapeutic targets of HCC were identified. These key targets were found to be frequently involved in 13 KEGG pathways and 4 biological processes. The expression of four representative key targets: TP53, CASP3, BCL2 and BAX further supports the suppression of TBM on HCC. In general, our study shows the curative effects of TBM against HCC. By using this integrative approach, we may find novel potential therapeutic targets to suppress HCC using TBM as an adjunctive therapy. And it could also help us understand the mechanism of HCC treatments in response to TBM.

## Introduction

Hepatocellular carcinoma (HCC) is a globally increased malignant cancer with high death occurrence (Jorge et al.; Elserag, 2011). The major management of HCC includes resection and chemotherapy (Baer et al., 1998; Whittaker et al., 2010). However, many drugs for HCC still have toxic side effects, which limit their usage in the clinic (Cheung et al., 2007; Sak, 2012; Chen et al., 2020; Zingales et al., 2020), and drug resistance of HCC hampers the treatment effects (Xiong et al.; Folmer et al., 2007; Tan et al., 2019; Li et al., 2020; Xu et al., 2020; Yang et al., 2020). Hence, it is urgent to discover new curative compounds with less adverse effects to treat this disease.

Traditional Chinese medicine (TCM) has been used to treat many diseases for more than 2500 years with its comprehensive active compounds and curative therapeutic effects. It has been reported that many herbs show suppressing effects on tumor cells (Wu and Yao; He, 2004; Dorsher and Peng, 2010). People have summarized Chinese herbal medicine’s advantages in reducing the side effects of chemo- and radiotherapy (Qi et al., 2010). Several formulas including astragalus, Turmeric (curcumin), and Ginseng have been used to reduce the toxicity as adjunctive therapies. For liver cancer treatment, formulations such as Bojungikki-tang, Kang-Fu-Zhi-Tong, PHY906, Xiao-Chai-Hu-Tang, Huang-Lian-Jie-Du-Tang, and Yin-Chen-Wu-Ling-San, have been reported to protect liver function, reduce cancer-related pain, improve respiratory tract infections and gastrointestinal side effects (Ling et al., 2007; Jeong et al., 2010; M. et al., 2010; Liu et al., 2011). *Bolbostemma paniculatum* (Maxim.) Franquet (Cucurbitaceae family), whose tuber named “Tu-Bei-Mu (TBM)” in Traditional Chinese Medicine, is one such herb (the name of the herb is verified in The Plant List (www.theplantlist.org) and Kew Medicinal Plant Names Services (https://mpns.science.kew.org/mpns-portal/)). Several groups reported the suppressing effects of TBM on several types of cancer (Islam et al.; Yu et al., 1992; Cheng et al., 2010; Xu, 2010; Chao et al., 2019). Thereby, we intend to study the effects of TBM on hepatocellular carcinoma and to decode the pharmacological mechanism of TBM’s action on HCC.

In 1987, a Japanese scientist Hiroko used the serum obtained from mice orally treated with the herbs to culture the cells. And the herb-contained serum effectively increased the mitogenic activity of lipopolysaccharide of murine splenic cells (Hiroko et al., 1987). Since then, many groups have been using herb-contained serum to treat the cells in order to study the herbs’ effect on diseases (Zhang et al., 2007; Rui et al., 2011). Cells directly treated by the herbs are easily disturbed by the chemical and physical properties of the herbs itself. Using serum from the rats orally treated or introgastrically administered with herbs can mimic the *in vivo* environment of the cell (Wang et al., 1999; Chen X. Z. et al., 2013). A serum complex containing the herb compounds, serum proteins, genes and other molecules takes its effect as a whole to the disease. The curative effects were obvious when cells were cultured with serum, orally treated or introgastrically administered with herbs (Yun-Ming, 2007; Chen L. et al., 2013). Thereby, we used the serum from rats introgastrically administered by TBM aqueous bulb extract to decipher its anti-tumor activity on the HCC cells.

As an alternative medicine remedy, TCM is characterized as multi-compound and multi-target, which hinders our understand the mechanism of the action. To address this problem, network pharmacology has been considered as a promising strategy. It combines experiments with bioinformatics, system biology and pharmacology to reveal the interactions among compounds, genes, proteins and metabolites (Lee; Hopkins, 2007; Da and Pei, 2014). This integrative approach helps us to more deeply understand the drug’s action on certain diseases and find potential curative compounds. In recent years, our group has been using this approach to decipher the pharmacological mechanism of the herb *Radix Ophiopogonis* in the treatment of Nasopharyngeal Carcinoma (Feng et al., 2019).

**Figure 1:**
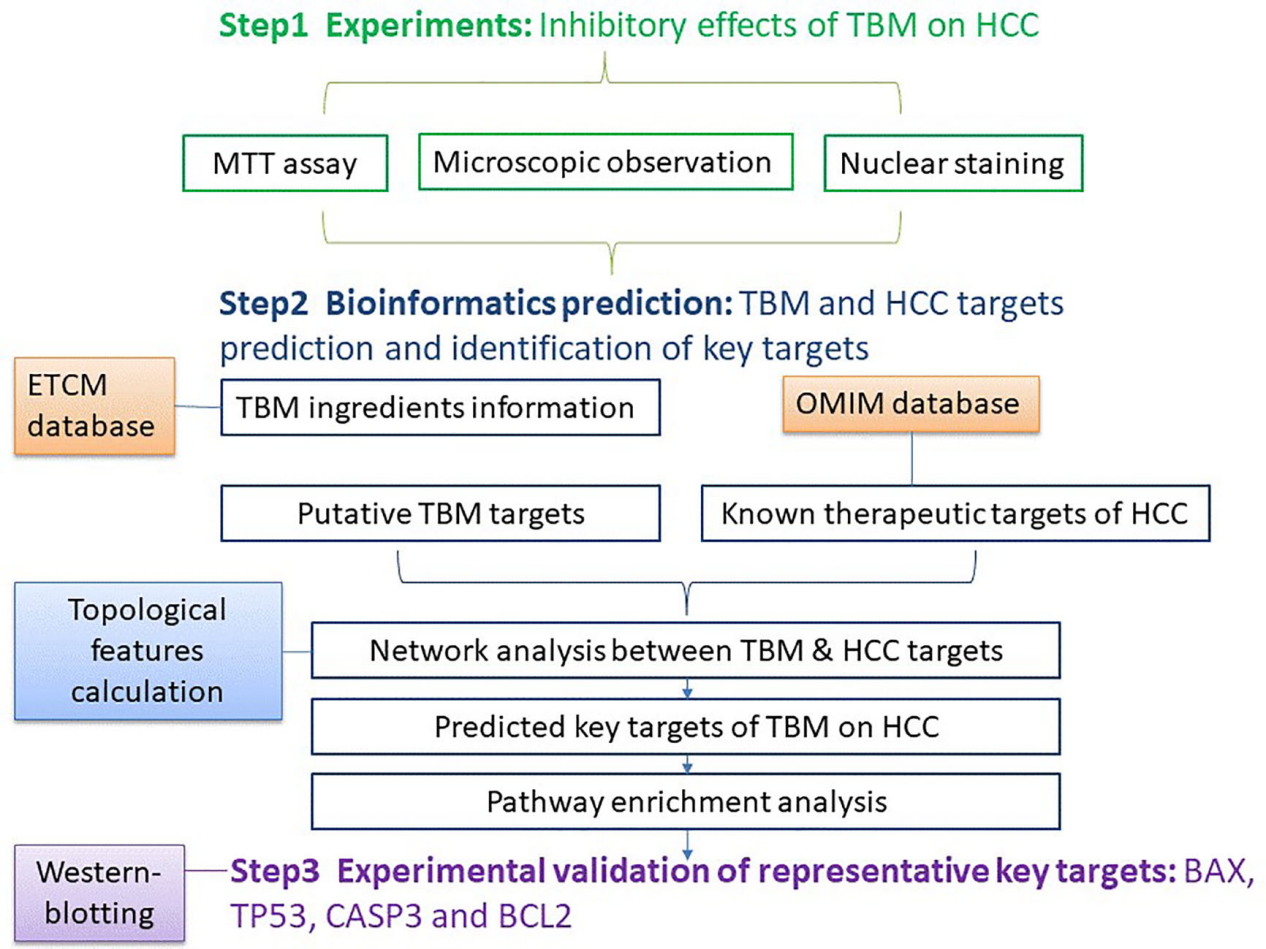
The work flow of studying the TBM’s inhibitory effects on HCC using an integrative pharmacology-based approach.

In this study, an integrative pharmacology-based approach was employed to understand the TBM’s effects on HCC by integrating experiments with bioinformatics predictions. Our study was implemented in three steps: (1) Lab experiments: Studying the effects of a TBM-contained serum on HCC; (2) Bioinformatics: TBM and HCC target prediction, network analysis and identification of key targets; (3) Validation of representative key targets of TBM against HCC. The workflow was elucidated in **Figure 1**.

## Material and Methods

### Step 1: Effects of TBM on HCC

#### Chemicals

RPMI-1640, 3-[4, 5-Dimethylthiazol-2-yl]-2, 5-diphenyltetrazolium bromide (MTT) powder, dimethyl sulfoxide (DMSO) (purity ≧99.7), anti-Bax, Bcl-2, p53, caspase-3 and β-actin primary antibodies were manufactured by Sigma-Aldrich Chemical Co., Ltd.

#### Preparation of the Fraction

The bulb of the *B. paniculatum* (TBM) was bought from the hospital of Shaanxi University of Chinese Medicine (Voucher number: 20181001). The TBM was cultivated and picked in Shaanxi province of China. The herbs were examined under the protocol of Chinese Pharmacopoeia (2015 vision). The TBM was characterized by Thin-layer chromatography (1.5% impurity, 11.9% H_2_O, 13mg/kg CO_2_ residue and 1.1% Tubeimuside I). The characterization and inspection were provided by Xing Sheng De pharmaceutical Co., Ltd (Report number: C201810070). The herb was further identified by Dr Bo Li (Shaanxi Collaborative Innovation Center of Chinese Medicinal Resources Industrialization, Shaanxi University of Chinese Medicine, China).200 ml distilled water was added to 60 g of coarsely powdered bulbs in a beaker. The mixture was kept in a water bath for 30min and boiled for 1h. The aqueous extract was then filtered and collected. The remaining powder was mixed with 200ml distilled water, boiled for 40min, filtered, and added to the prior filtrate. After evaporation, the raw concentration of the TBM was 2.5g/ml. The TBM mixture was stored at 4°C in labelled sample bottles. We heated the mixture to 37°C when it wass needed to feed the rats.

#### Animals and Treatments

30 adult female Wistar rats (200–220 g) were purchased from the Experimental Animal Center of Xi’an Jiaotong University (license number: SCXK 2017-003). The animals were housed (5 rats/cage) in the standard rats plexiglass cages in a room maintained under standard conditions of temperature and humidity with an alternating 12-hr light/dark cycle and we provided regular commercial pellet diet and water to the rats. Female Wistar rats were randomly divided into two groups: the control group and the TBM group. Control group (n = 15) received 2ml tap water twice per day for 14 days by intragastric administration. TBM group (n = 15) received the aqueous fraction of TBM with the same frequency. One hour after the last time intragastric administration, all rats were anesthetized with 2% halothane in 100% O_2_ blown over the nose through a face mask. Then the blood was collected from the abdominal aorta of the rats. The serum was collected after centrifuging the blood for 10min at 3000r/min and stored at -20°C for use. To ensure the biosecurity, we all follow the standard protocols in each step. All experiments were conducted in accordance with the ethical guidelines of the Declaration of Helsinki, and all experimental procedures were approved and performed in accordance with the guidelines of the local Institutional Animal Experimentation Ethics Committee.

#### Cell Culture

The human hepatocellular carcinoma cell line BEL-7404 and the human liver cell line L-02 were purchased from the Cell Bank of the Type Culture Collection of the Chinese Academy of Sciences (Shanghai, China) and cultured in the RPMI-1640 containing 10% (v/v) heat-inactivated fetal bovine serum (FBS), 100 μg/ml streptomycin and 100 U/ml penicillin at 37°C in a 5% CO_2_ incubator. After the cells were grown into confluency, the HCC BEL-7404 cell line was divided into the following 4 groups: 10% fetal bovine serum (Contro), 25% TBM serum, 12.5% TBM serum and 6.25% TBM serum respectively prior to MTT assay and nuclear staining (The original serum obtained from rats containing TBM was considered as 100% TBM serum). The human liver cell line L-02 were regrouped into 4 groups with the same serum accordingly (Control, 25% TBM, 12.5% TBM and 6.25% TBM respectively.)

#### MTT Assay

The MTT (3-(4,5-dimethylthiazol-2-yl)-2,5-diphenyltetrazolium bromide) assay was performed with 1×10^4^ cells/well in 96-well plates. After culture for 12h, the cells were incubated with serum containing TBM. Then, 20 μl MTT (5 mg/ml) was added for 4 h. After removal of the medium, the formazan crystals were dissolved in 150 μl DMSO. Absorbance was obtained at 570 nm with an ELx-800 Universal Microplate Reader (BioTek, Winooski, VT). Cell Inhibition Ratio (%) (IR) was determined as follows:

Inhibition Ratio (%) = [(ODcontrol − ODtreated)/(ODcontrol − ODblank)] ×100% (The control was 10% FBS serum; the treated cells are HCC cell line BEL-7404 and the human liver cell line L-02 treated by different concentrations of TBM; the blank was DMSO)

#### Nuclear Staining

BEL-7404 cells treated with the serum containing TBM were fixed with paraformaldehyde at room temperature for 10 min. Then the cells were incubated with 20 µl DAPI at a concentration of 10 µg/ml (10 min, room temperature). Nuclear morphology was observed using a fluorescence microscope (Olympus FV1000).

#### Statistical Analysis

We display the data as Mean ± SD from 3 independent experiments. A student’s t-test was employed to test the deviation between different batches. Values were only considered to be statistically significant when p-values were less than 0.05.

### Step 2: TBM and HCC Target Prediction, Network Analysis and Identification of Key Targets

#### Compositive Compounds of TBM

The chemical compounds of TBM were obtained from The Encyclopedia of Traditional Chinese Medicine (ETCM) database (Huang et al., 2015; Haiyu et al., 2016; Yu et al., 2017; Xu et al., 2018) (http://www.nrc.ac.cn:9090/ETCM/index.php/Home/, updated on 2018). The chemical compounds contained in the TBM aqueous bulb extract were selected by the criteria “Tanimoto scores > 0.8.” This score represents the similarity degree of the certain chemical components to that of known drugs. Tanimoto score ranges from “0 –1”, where “0” means the completely different structures between ingredients and known drugs, and “1” means the same structures of two compounds. This Tanimoto score is the criteria to remove redundant compounds. The known compounds with high structural similarity (the structural similarity score is higher than 0.8) were identified as the putative compounds of TBM. The compounds with a similarity score lower than 0.8 were seen as redundant compounds. Under the criteria above, 10 chemical compounds were obtained from TBM. Detailed information of the chemical compounds contained in TBM is provided in **Supplementary Table S1**.

#### TBM Putative Targets Prediction

The known putative targets of TBM were identified from the ETCM database as described above (Xue et al., 2013). As described above, putative targets of certain chemical components were predicted by comparing the structure of the certain chemical components to that of known drugs. The targets of known drugs with high structural similarity (the structural similarity score is higher than 0.8) were identified as the putative targets of the certain chemical components. After removing redundancy, we found 155 TBM putative gene targets. These targets are also provided in **Supplementary Table S1**.

#### Known Therapeutic Targets of HCC

Known therapeutic targets for the treatment of HCC were collected from the OMIM database (www.omim.org, last updated October 31, 2013) (Hamosh et al., 2005). Only Food and Drug Administration (FDA) approved drugs used in humans were chosen. In total, 282 therapeutic targets were selected as HCC-related targets. Detailed information on the collected known therapeutic targets is provided in **Supplementary Table S2**.

#### Pathway Enrichment Analysis of TBM and HCC Putative Targets

Both TBM putative targets and known therapeutic targets of HCC treatments were analyzed through pathway enrichment analysis. We used the Database for Annotation, Visualization and Integrated Discovery (DAVID, http://david.abcc.ncifcrf.gov/home.jsp, version 6.8) and Kyoto Encyclopedia of Genes and Genomes database (KEGG, http://www.genome.jp/kegg/, updated on April 18, 2016) (Kanehisa and Goto, 2000; Dennis et al., 2003) to analyze those targets and involved pathways.

#### Network Analysis Between TBM Putative Targets and HCC Known Therapeutic Targets

We use the STRING (Search Tool for the Retrieval of Interacting Genes/Proteins, https://string-db.org/, vision 10.5) database to analyze the interaction between TBM putative targets and HCC known therapeutic targets. Interaction confidence of proteins were indicated by scores. We collected the targets when the combined score of those were higher than the medium value. Targets with the combined score lower than the medium value were discarded. Detailed information obtained is provided in **Supplementary Table S3**. Then we use Cytoscape (Vision 3.5.1, Boston, MA, USA) to visualize the networks.

#### Identification of Key Predicted Targets Based on TBM-HCC Interaction Network

Based on the above network, we further identified the major nodes in the direct protein-protein interactions. We employ the three topological features: “degree”, “betweenness” and “closeness” as criteria to screen the putative targets due to their topological importance. “Degree” was defined as the number of links to one node. “Node betweenness” was defined as the number of the shortest paths between pairs of nodes that ran through one node. “Closeness” was defined as the inverse of the farness, which was the sum of the node distances to all other nodes. The closeness centrality can be regarded as a measure of how long it will take to sequentially spread information from the node to all the other nodes. Degree, node betweenness and closeness centralities can be used to measure a protein’s topological importance in the network. A larger value of a certain protein’s degree/node betweenness/closeness centrality indicates that this protein is more important in the network (Wang et al., 2012). Only nodes with topological features higher than the corresponding median values were identified as the major targets. After screening with the above criteria, 66 candidates were identified as key targets, provided in **Supplementary Table S4**.

### Step 3: Validation of Representative Key Targets of TBM Against HCC

#### Western-Blotting of Key Targets

On the spectrum of key targets’ biological and topological significance, we chose caspase-3, p53, Bcl-2 and Bax to detect their expression in TBM-treated cells. As described previously, the HCC cell line BEL-7404 was treated with 10% fetal bovine serum (Contro), 25% TBM serum, 12.5% TBM serum and 6.25% TBM serum respectively. When the cells were grown into 1×10^6^ per plate, we treated the cell line with 2, 4 or 6 μg/ml of peiminine for 24 h. Cell lysates were prepared in RIPA buffer (50 mmol/L Tris-HCl buffer, pH 7.4, 150 mmol/L NaCl, 1% Triton X-100, 1% sodium deoxycholate, and 0.1% sodium dodecyl sulfate) supplemented with a protease inhibitor to prevent the protein degradation. Cell lysates were centrifuged at 12,000×g for 15 min at 4°C. Total protein was extracted from the resulting supernatant and the concentration was quantified by the BCA assay. Proteins were separated on 10-12% sodium dodecyl sulfate-polyacrylamide gel electrophoresis and transferred to PVDF membranes. The membranes were firstly treated overnight at 4°C with rabbit monoclonal anti-human β-actin, caspase-3, p53, Bcl-2 and Bax primary antibodies. And then the membranes were washed with PBS incubated with appropriate horseradish peroxidase (HRP)-conjugated secondary antibodies for 1 hour at room temperature. The chemiluminescence of proteins transferred to PVDF membranes was detected with ECL kit (GE Healthcare Amersham, Piscataway, NJ).

## Results

The work-flow of studying the TBM’s inhibitory effects on HCC using an integrative pharmacology-based approach was elucidated in **Figure 1**.

### Step I: Inhibitory Effects of TBM on HCC

#### Cytotoxicity of TBM on HCC

**Figure 2:**
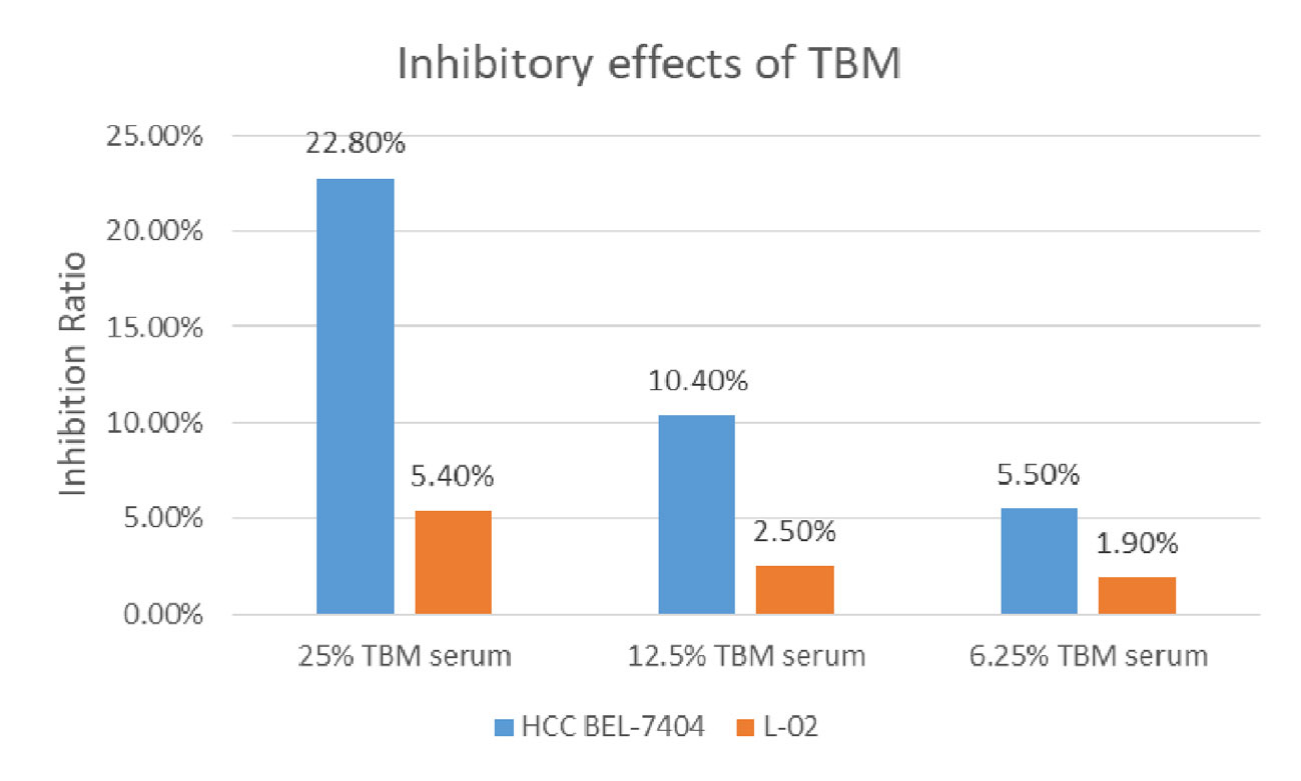
Inhibition of the TBM serum on the HCC cell line BEL-7404 and the human liver cell line L-02. The Inhibition ratio was calculated as follows: Inhibition Ratio (%) = [(ODcontrol − ODtreated)/(ODcontrol − ODblank)] ×100%. The control was 10% FBS serum. The blue bar shows the inhibition ratio of the TBM serum with different concentrations upon HCC BEL-7404 cells and the yellow bar shows the inhibition ratio of the TBM serum upon the human liver cell line L-02.

The cytotoxicity of the serum containing the TBM aqueous bulb extract upon HCC cell line BEL-7404 and the human liver cell line L-02 was assessed by the MTT assay. We considered the original serum from the rats treated with TCM as 100% TBM serum. We observed the cell viability after 24h and 48hrs. We chose 25%, 12.5% and 6.25% TBM serum to treat the cells. 10% FBS was used as the control medium. As shown in **Table 1A**, the OD values were significantly decreased after the HCC cells were treated with the TBM serum. The 25% TBM serum inhibited the HCC cells with the inhibition ratio 22.8%, indicating that TBM can significantly inhibit the growth of the HCC cells. Meanwhile, the TBM serum has much fewer inhibitory effects on the human liver cell line L-02 as shown in **Table 1B**. The total inhibitory effects were shown in **Figure 2**.

**Table 1A:** The inhibitory effects of the TBM serum on the HCC cell line BEL-7404.

	OD	Inhibition Ratio
**Control**	0.5891 ± 0.0061	0
**25% TBM serum**	0.4550 ± 0.0089	22.8%
**12.5% TBM serum**	0.5278 ± 0.0162	10.4%
**6.25% TBM serum**	0.5596 ± 0.0158	5.5%

The control was 10% FBS serum. Data are presented as the mean ± SD from three independent experiments(p < 0.05).

**Table 1B:** The inhibitory effects of the TBM serum on the human liver cell line L-02.

	OD	Inhibition Ratio
**Control**	0.4891 ± 0.0055	0
**25% TBM serum**	0.4633 ± 0.0146	5.4%
**12.5% TBM serum**	0.4775 ± 0.0127	2.5%
**6.25% TBM serum**	0.4823 ± 0.0166	1.9%

The control was 10% FBS serum. Data are presented as the mean ± SD from three independent experiments(p < 0.05).

#### Microscopic Observation Showing TBM’s Inhibition on HCC Cell Line BEL-7404 With Less Cytotoxicity Towards the Human Liver Cell Line L-02

**Figure 3:**
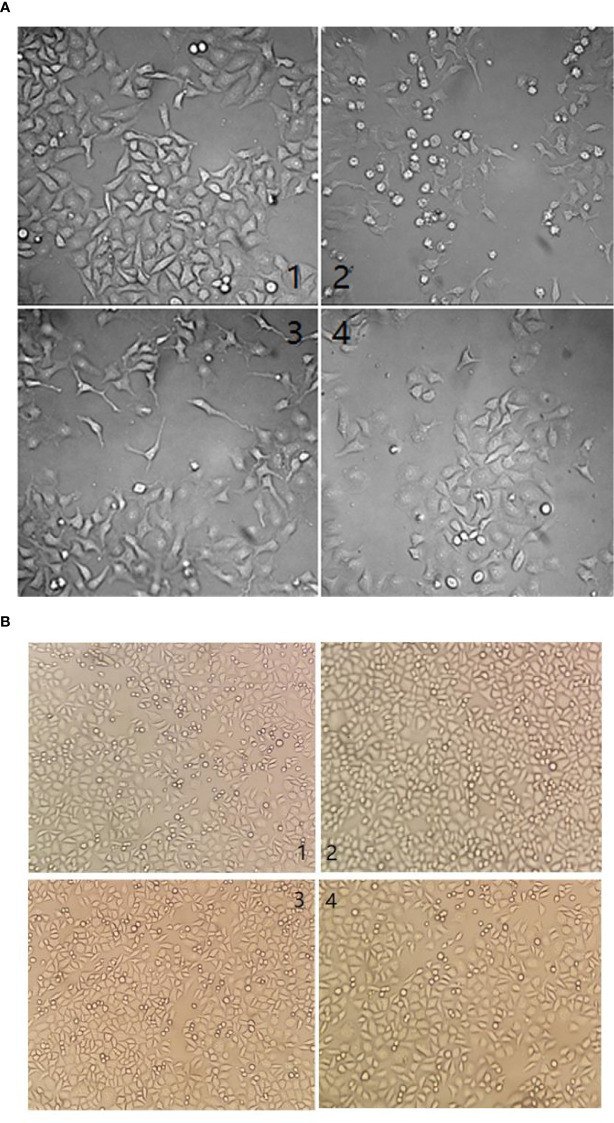
**(A)** Morphological changes by different serum treatments on the HCC cell line BEL-7404 after 48h. (1) cells were cultured with 10% FBS at a concentration of 5.1 × 10^7^ cfu/ml. (2) Cells were cultured with 25% TBM serum at the same concentration. (3) Cells were cultured with 12.5% TBM serum at the same concentration. (4). Cells were cultured with 6.25% TBM serum at the same concentration. **(B)** Morphological changes by different serum treatment on the human liver cell line L-02 after 48h. (1) cells were cultured with 10% FBS at a concentration of 5.1 × 10^7^ cfu/ml. (2) Cells were cultured with 25% TBM serum at the same concentration. (3) Cells were cultured with the 12.5% TBM serum at the same concentration. (4). Cells were cultured with 6.25% TBM serum at the same concentration.

Representative images of the morphological changes observed after 48h are shown in **Figures 3A**, **B**. Compared to the control (FBS-treated cells), the HCC cell line BEL-7404 exposed to the TBM serum was significantly inhibited (**Figure 3A**). And the human liver cell line L-02 showed no obvious difference when they were treated with the TBM serum (**Figure 3B**).

#### Nuclear Morphology of the HCC Cell Line BEL-7404 Treated With the TBM Serum

**Figure 4:**
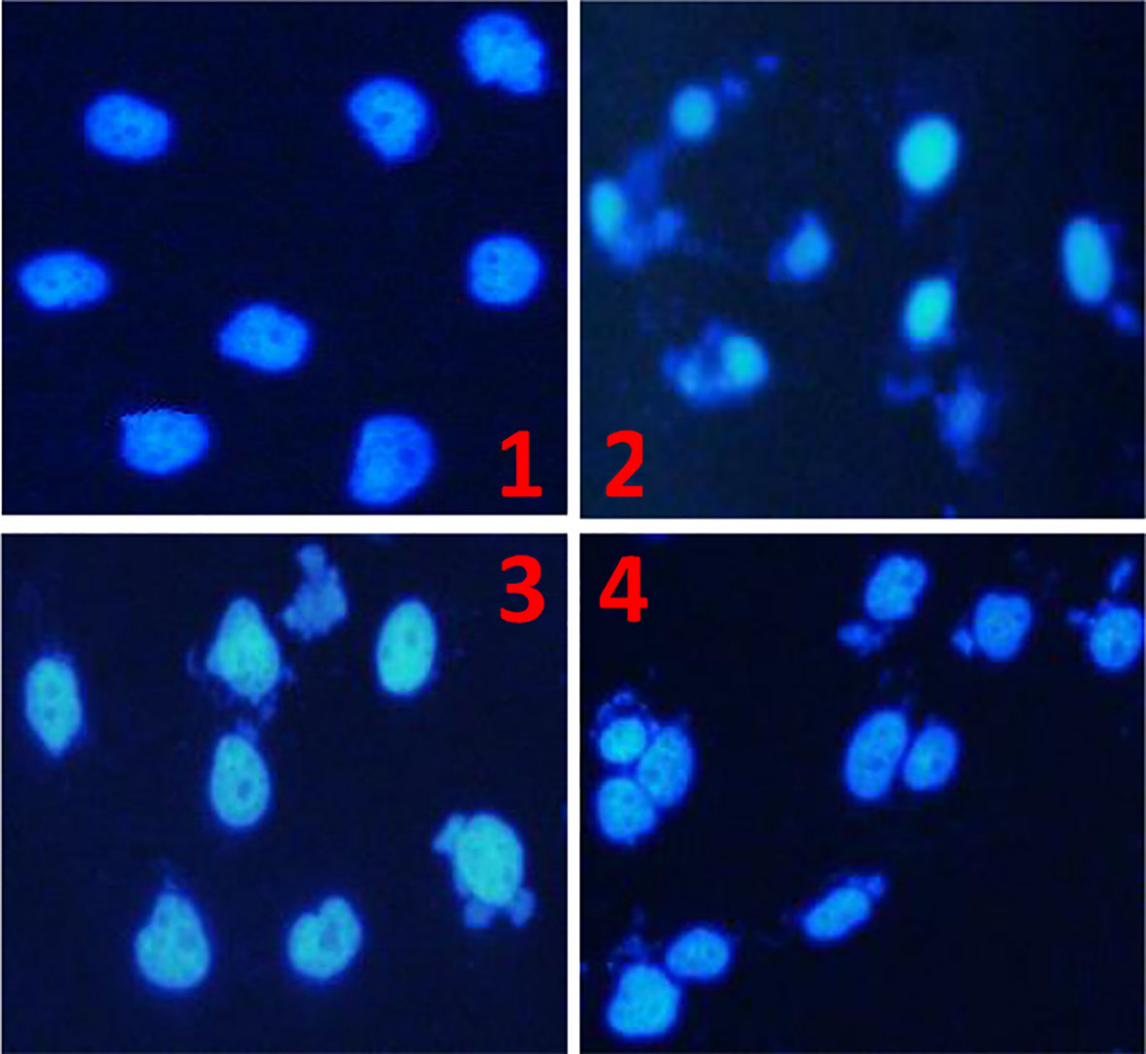
The Morphology of the HCC cell line BEL-7404 by nuclear staining. The HCC BEL-7404 cells were stained using DAPI solution and observed by fluorescence microscopy (×400). (1) The HCC cells cultured with 10% FBS serum for 48h (Control). (2) The HCC cells cultured with 25% TBM. (3) The HCC cells cultured with 12.5% TBM. (4) The HCC cells cultured with 6.25% TBM.

The results of nuclear staining further confirmed that TBM could inhibit the growth of HCC cells. After culturing the HCC cell line BEL-7404 with 10% FBS serum (control), 25% TBM, 12.5% TBM and 6.25% TBM respectively for 48h, the cells show different nuclear morphology as elucidated in **Figure 4**. Compared with the control, the nuclear morphology of the cells treated with TBM were damaged obviously. A higher concentration of TBM culture resulted in more cell damage.

### Step 2: Bioinformatics Prediction

#### Chemical Compounds of TBM and Its Potential Gene Targets Prediction

The ETCM database was employed to obtain the chemical compounds of TBM as described previously. As a result, we collected 10 chemical compounds contained in TBM under the criteria “Tanimoto score > 0.8” as described previously in **Supplementary Table S1**. In total, 155 putative TBM gene targets were collected, also shown in **Supplementary Table S1**.

#### TBM Compositive Compound-Putative Target Network

**Figure 5:**
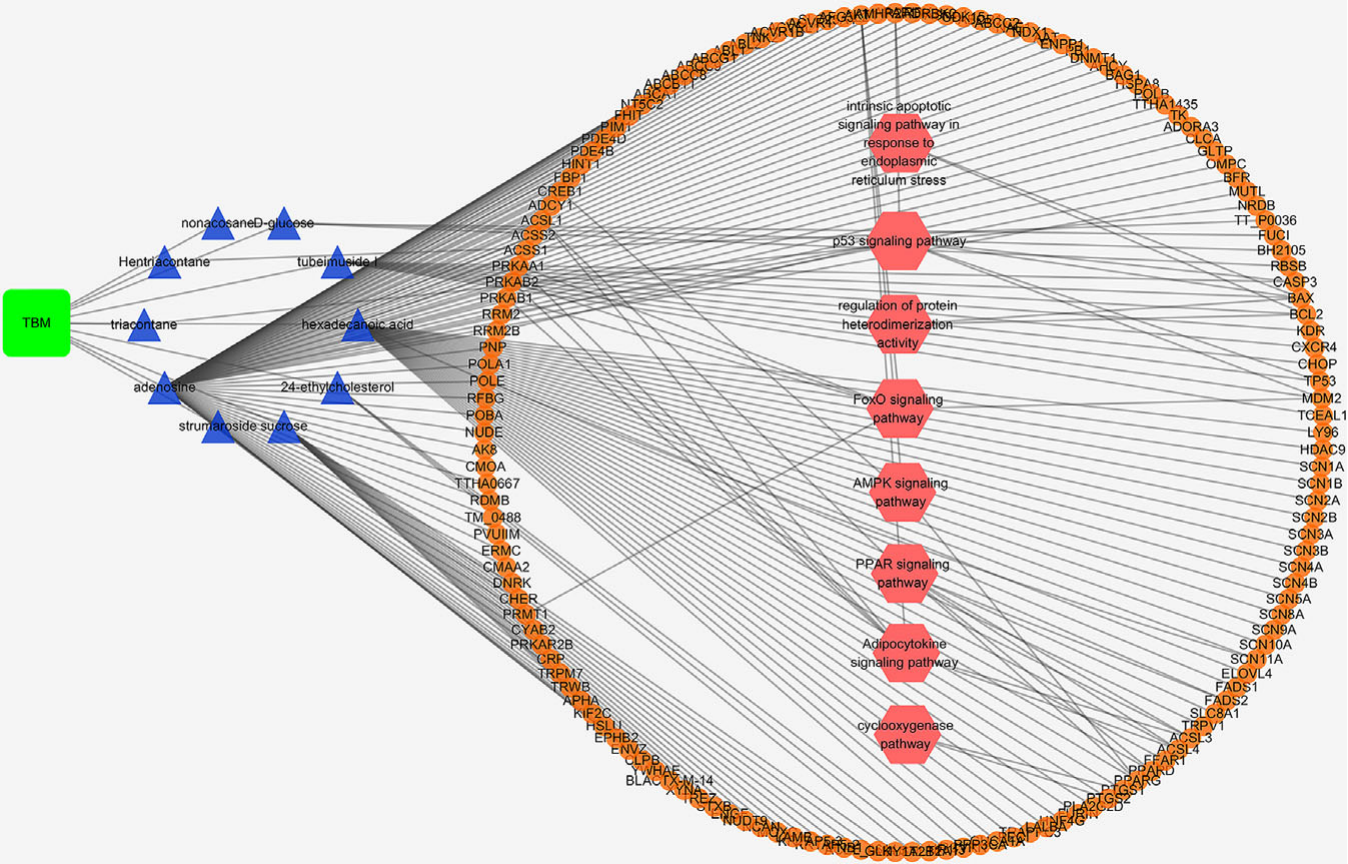
TBM compositive compound-putative target network built and visualized with Cytoscape. Edges: interactions between compositive compounds of TBM and their putative targets; green square node: the herb TBM; blue triangular nodes: compositive compounds of TBM; orange round nodes: putative targets of compositive compounds of TBM; pink hexagonal nodes: the main pathways from enrichment analysis of major targets.

A TBM compositive compound-putative target network was built to understand the interaction between the TBM’s ingredients with its putative targets as indicated in **Figure 5**. The network consists of 166 nodes (1 herb, 10 compositive compounds and 155 putative targets) and 165 edges. 91 genes with a combined score higher than the medium value were selected as the major TBM putative targets. By pathway enrichment analysis, we found these TBM putative targets were frequently involved in 8 pathways and biological processes including the p53 signaling pathway, Adipocytokine signaling pathway, PPAR signaling pathway, AMPK signaling pathway, FoxO signaling pathway, cyclooxygenase pathway, intrinsic apoptotic signaling pathway in response to endoplasmic reticulum stress and regulation of protein heterodimerization activity.

#### TBM Compositive Compound-Putative Known HCC Target Network

**Figure 6:**
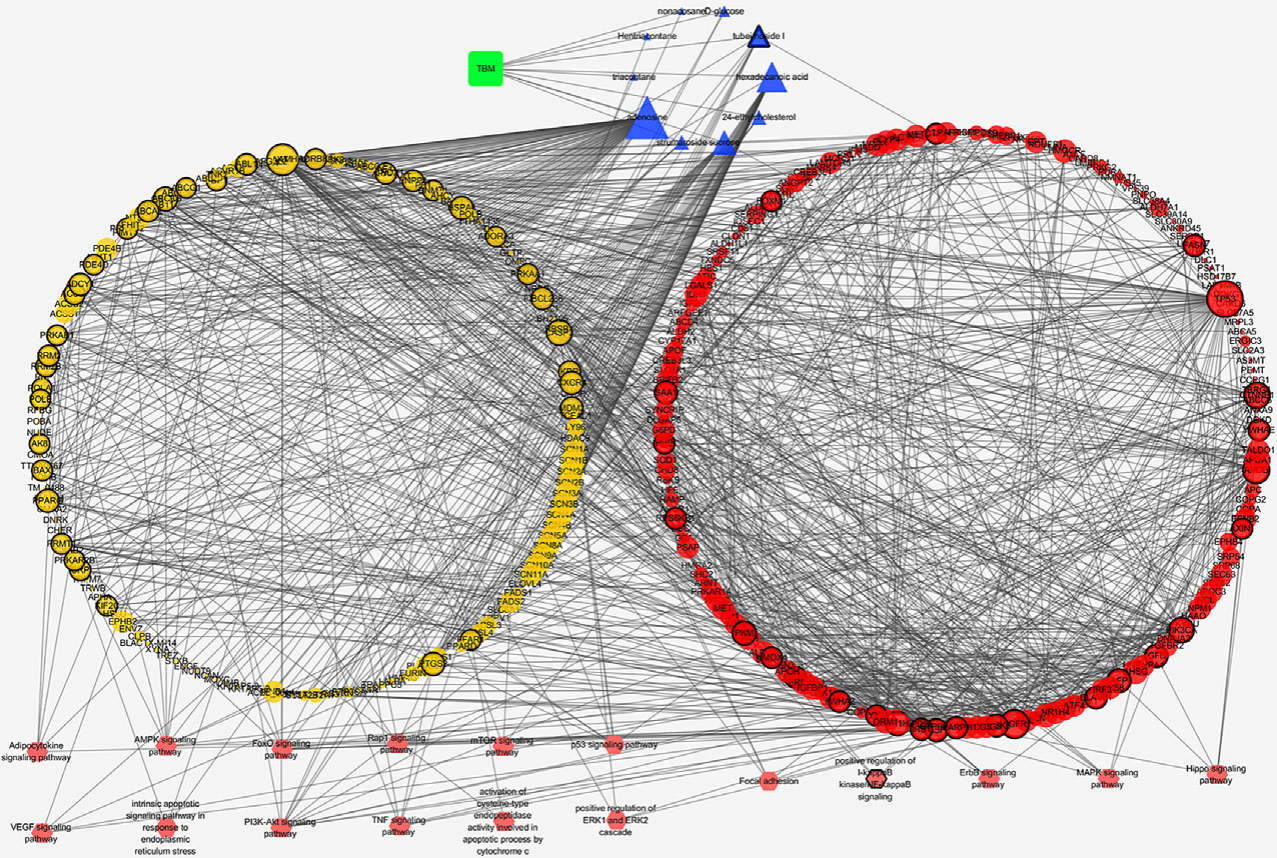
TBM compositive compound-putative known HCC target network visualized with Cytoscape. Edges: Interactions among TBM, compositive compounds, putative targets, and known therapeutic targets of HCC. Green square nodes: the ingredient TBM; blue triangular nodes: compositive compounds of TBM; yellow round nodes: putative targets of TBM compositive compounds; red round nodes: known therapeutic targets for the treatment of HCC; pink hexagonal nodes: the pathways from enrichment analysis of key nodes. Nodes marked with dark rings: key nodes (the “degree,” “node betweenness,” and “closeness” of which were all larger than the corresponding median values).

We analyzed the interaction between the TBM compositive compounds’ targets and putative known HCC targets to evaluate the effects of TBM on HCC. The interaction data of TBM putative targets and HCC known therapeutic targets was acquired as previously described in the materials and methods section. As shown in **Figure 6**, the network consists of 307 nodes (containing 1 ingredient, 10 compositive compounds of TBM, 155 TBM putative targets, and 282 known therapeutics of HCC) and 1086 edges. As mentioned in material and methods section, three topological features “degree”, “betweenness” and “closeness” were employed as criteria to screen the putative targets for topological importance. Only nodes with topological features higher than the corresponding median values were identified as major nodes. As a result, we identified 66 major nodes containing 1 ingredient, 3 compositive compounds in HCC, 36 TBM’s putative targets and 26 known therapeutic targets for the treatments of HCC. Details concerning the topological features of the 66 major nodes in this network was shown in **Supplementary Table S4**. Except the ingredient TBM and its three compounds, 62 gene targets (36 TBM’s targets and 26 known therapeutic targets of HCC) were considered as the key TBM-HCC predicted targets. According to the pathway enrichment analysis based on the GO annotation system and the KEGG pathway, the key TBM-HCC targets were frequently involved in 4 biological processes and 13 KEGG pathways as shown in **Table 2**. Based on the number of the edges between key targets and pathways, TBM’s key targets were found to be strongly connected with Adipocytokine signaling pathway, AMPK signaling pathway, FoxO signaling pathway, Rap1 signaling pathway, mROT signaling pathway, p53 signaling pathway, VEGF signaling pathway, intrinsic apoptotic signaling pathway in response to endoplasmic reticulum stress, PI3K-AKT signaling pathway, TNF signaling pathway, activation of cysteine-type endopeptidase activity involved in apoptotic process by cytochrome c and positive regulation of ERK1 and ERK2 cascade. In the meantime, HCC key gene targets indicate that they are frequently involved in focal adhesion, positive regulation of I-kappaB kinase/NF-kappaB signaling, ErbB signaling pathway, MAPK signaling pathway and Hippo signaling pathway.

**Table 2:** Major GO biological processes and KEGG pathways with their key targets.

Term	Key targets	P-value
Biological processes		
GO:0070059 intrinsic apoptotic signaling pathway in response to endoplasmic reticulum stress	BCL2, BAX, APAF1	0.0059
GO:0008635 activation of cysteine-type endopeptidase activity involved in apoptotic process by cytochrome c	BAX, APAF1	0.0184
GO:0043123 positive regulation of I-kappaB kinase/NF-kappaB signaling	HMOX1, CASP8, ABL1, CTNNB1	0.0192
GO:0070374 positive regulation of ERK1 and ERK2 cascade	EGFR, ABL1, TGFB1, KDR	0.0209
**KEGG pathways**		
cfa04152:AMPK signaling pathway	AKT1, CREB1, PPARG, PRKAB2, FBP1, PRKAB1, FASN, PIK3CA, ELAVL1, RPS6KB1, PRKAA1, IRS1	1.71E-09
cfa04068:FoxO signaling pathway	EGFR, AKT1, PRMT1, MAPK14, PRKAB2, PRKAB1, PIK3CA, MDM2, PRKAA1, IRS1, TGFB1	5.16E-08
cfa04115:p53 signaling pathway	CASP3, RRM2, BAX, CASP8, TP53, MDM2, RRM2B, APAF1	6.00E-07
cfa04151:PI3K-Akt signaling pathway	EGFR, AKT1, YWHAZ, BCL2, CREB1, TP53, MDM2, PIK3CA, RPS6KB1, PRKAA1, IRS1, YWHAE, KDR	8.24E-06
cfa04668:TNF signaling pathway	AKT1, CASP3, PTGS2, MAPK14, CREB1, CASP8, PIK3CA	1.40E-04
ptr04151:PI3K-Akt signaling pathway	AKT1, CREB1, VEGFA, TP53	0.0107
cfa04920:Adipocytokine signaling pathway	AKT1, ACSL1, PRKAB2, PRKAB1, PRKAA1, IRS	2.17E-04
cfa04370:VEGF signaling pathway	AKT1, PTGS2, MAPK14, PIK3CA, KDR	0.0011
cfa04150:mTOR signaling pathway	AKT1, PIK3CA, RPS6KB1, PRKAA1, IRS1	0.0011
cfa04012:ErbB signaling pathway	EGFR, AKT1, PIK3CA, RPS6KB1, ABL1	0.0047
cfa04015:Rap1 signaling pathway	EGFR, AKT1, ADCY1, MAPK14, PIK3CA, KDR, CTNNB1	0.0053
cfa04390:Hippo signaling pathway	AFP, YWHAZ, YWHAE, TGFB1, CTNNB1, AXIN1	0.0063
cfa04010:MAPK signaling pathway	EGFR, AKT1, CASP3, MAPK14, TP53, TGFB1, HSPA8	0.0125
cfa04510:Focal adhesion	EGFR, AKT1, BCL2, PIK3CA, KDR, CTNNB1	0.0225

#### Step 3: Expression of Representative Key Targets Validated by Western Blotting

**Figure 7:**
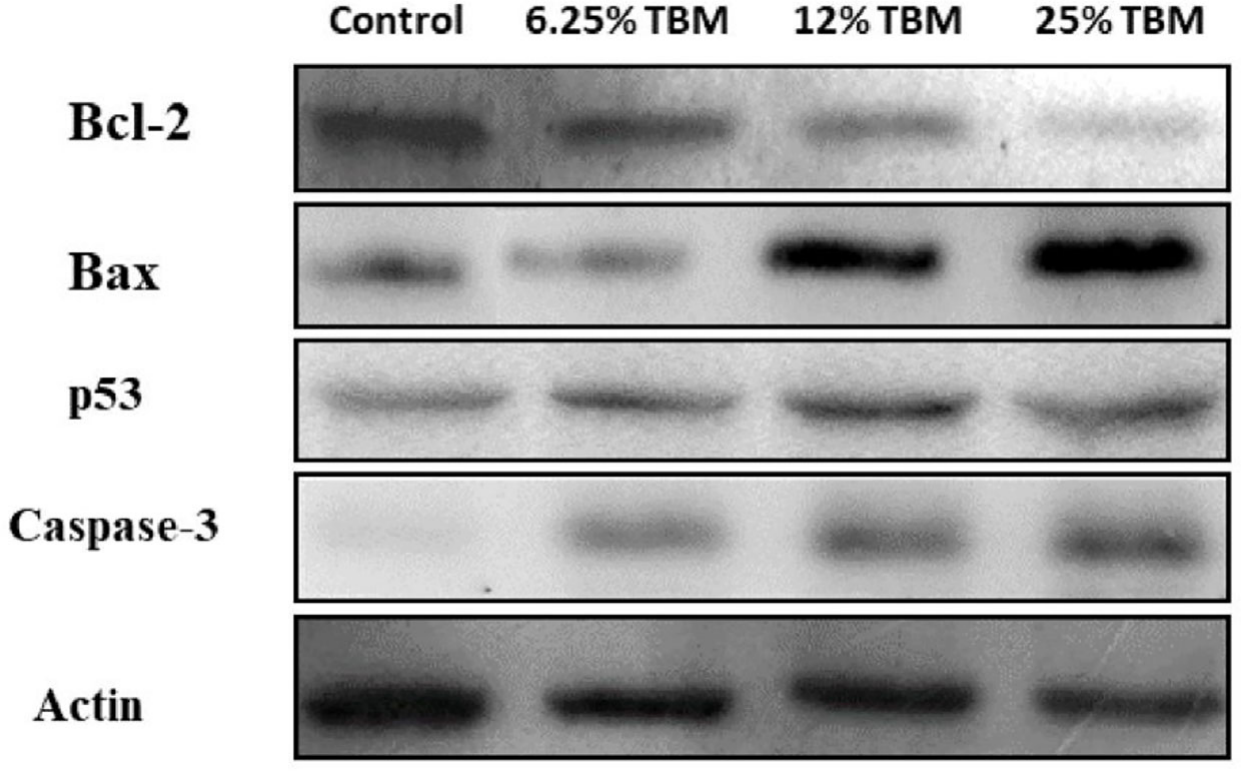
Expression of Bcl-2, Bax, p53 and Caspase-3 in TBM-treated HCC cells validated by Western blotting. The Control group is the expression of representative key targets when cells are cultured with 10% FBS. The other three groups are the expression of these targets when cells are cultured with 6.25%, 12.5% and 25% TBM serum respectively (*p* < 0.05).

In total, 62 gene targets were selected as key TBM-HCC targets. These targets show an important role potentially during TBM-HCC interactions. Based on the three topological features “degree”, “betweenness” and “closeness”, the “degree” ranges from 6-61, the “betweenness” ranges from 4.55x10^-4^ to 0.1279, the “closeness” ranges from 0.3252 to 0.4287, we chose four key targets averagely distributed on the spectrum of their topological significance to validate their expression by Western blotting. The topological values of these four key targets were as follows: TP53 degree 61, betweenness 0.1278, closeness 0.4129; CASP3 degree 24, betweenness 0.0246, closeness 0.3711; BCL2 degree 12, betweenness 0.0028, closeness 0.3333; BAX degree 8, betweenness 4.55x10-4, closeness 0.3271; The detailed information of the key targets is provided in **Supplementary Table S4**. As shown in **Figure 7**, the expression of TP53, CASP3, and BAX were significantly increased when HCC cells were treated with the aqueous extract of TBM. BCL2 was down-regulated when cells were treated by 12%TBM and 25% TBM. The tumor suppressor TP53, CASP3 and BAX genes were mostly down-regulated in many types of tumors including liver cancer, while BCL2 gene suppresses the apoptosis. The up-regulation of TP53, CASP3 and BAX and down-regulation of BCL2 supports the TBM’s suppression effect on HCC cells. This result further confirms that the TBM’s aqueous bulb extract is effective to suppress the growth of HCC cells by regulating these key targets.

## Discussion

Traditional Chinese medicine has been proved to treat cancer effectively with its multi-target therapeutics. TBM, characterized with multi-components and multi-targets, have been used to suppress liver cancer as an adjunctive therapy in recent years. In this study, we develop an integrative pharmacology-based strategy to decipher the mechanism of the TBM’s aqueous bulb extract in suppressing HCC. By integrating experiments, bioinformatics prediction and validation, 62 key targets (36 TBM’s targets and 26 known therapeutic targets of HCC) were identified after analyzing TBM-HCC interactions. These key targets were frequently involved in 13 KEGG pathways and 4 biological processes. The integrative analysis bridging experiments with bioinformatics prediction provides us novel potential therapeutic targets to treat HCC using TBM. And it could also help us understand the mechanism of HCC treatments in response to TBM.

All 62 key targets have shown their topological and biological significance (**Table 2**). On the topological spectrum of these key targets, we choose 4 representative targets because they are averagely distributed on the topological table (**Supplementary Table S4**). The degree score of TP53, CASP3, BAX and BCL2 are 61, 24, 8 and 12 respectively (the total degree ranges from 6 to 61).

Tumor suppressor protein p53 (TP53) is a suppressor of tumor cells by inducing growth arrest or apoptosis (Moshe et al., 2002). This study revealed that TP53 were frequently involved in the p53 signaling pathway, PI3K-Akt signaling pathway and MAPK signaling pathway in hepatocellular carcinoma. TP53 takes important roles in the signaling pathways of HCC (Sia and Villanueva; Villanueva et al.; Whittaker et al., 2010). Repressed by MDM2, TP53 takes effects on cell survival in PI3K-Akt signaling pathway (Luo et al., 2003; Vara et al., 2004; Gang et al., 2005). the crosstalk between p53 protein and the MKK3/MKK6/p38 MAPK Signaling Pathway in Cancer has also been reported (Lorenzo et al.). The up-regulation of TP53 in the TCM-treated cells indicates TCM’s inhibitory effects on tumor cells. And the p53 signaling pathway could act as a significant route when the curative effect of TCM takes place on HCC.

CASP3 encodes a cysteine-aspartic acid protease that plays a central role in the execution-phase of cell apoptosis. We found CASP3 was frequently involved in p53 signaling pathway, TNF signaling pathway and MAPK signaling pathway in this study. This gene has been recognized for its importance in regulating hepatocellular carcinoma (Aquino et al.; Lu et al.). Our study shows the TBM’s action on HCC cells lead to the down-regulation of CASP3, suggesting CASP3’s critical role during treating HCC with TBM.

BAX encodes Bax protein which forms a heterodimer with BCL2, and functions as an apoptotic activator. The association and the ratio of BAX to BCL2 also determines survival or death of a cell following an apoptotic stimulus. Compared with normal cells, the tumor cells always show a decreased expression of the Bax/BCL2 ratio (Sakinah et al., 2007; Wei et al., 2008). Our study shows an up-regulation of BAX and a down-regulation of BCL2 after the HCC cells were cultured with the aqueous extract of TBM. This result suggests TBM might take its inhibitory effect on HCC *via* regulating the Bax/BCL2 ratio. Furthermore, the result of Western blotting indicates higher concentration of TBM (12% and 25% TBM) is more effective to increase the expression of BAX and to decrease the expression of BCL2.

## Conclusion

This study, for the first time, reports that TBM-containing serum could suppress the growth of HCC cells. And we employ an integrative pharmacology-based strategy combining experiments and TCM-HCC network analysis to decipher the mechanism behind. From the results, the inhibitory effects of TBM might be related to its regulation on 62 key targets *via* 13 KEGG pathways and 4 biological processes. Our findings could help us consider novel potential gene targets when treating the hepatocellular carcinoma and understanding the mechanism of curative effect of TBM.

## Data Availability Statement

All datasets presented in this study are included in the article/**Supplementary Material**.

## Ethics Statement

The animal study was reviewed and approved by Shaanxi University of Chinese Medicine Animal Experimentation Ethics Committee.

## Author Contributions

XF and XC designed the research. XF performed the research and wrote the paper. GM cultured the cells and feed the rats. HS contributed analytic tools. YW carried out the experimental validation. All authors contributed to the article and approved the submitted version.

## Funding

This study was supported by the fund from the Subject Innovation Team of Shaanxi University of Chinese Medicine (2019YS05) and Basic Scientific Research Project of Shaanxi Province (2020JQ-867).

## Conflict of Interest

The authors declare that the research was conducted in the absence of any commercial or financial relationships that could be construed as a potential conflict of interest.
