# Corrigendum: An integrative serum pharmacology-based approach to study the anti-tumor activity of B. Paniculatum aqueous bulb extract on the human hepatocellular carcinoma cell line BEL-7404

**DOI:** 10.3389/fphar.2022.1054143

**Published:** 2022-11-08

**Authors:** Xuesong Feng, Guangyuan Ma, Hailong Shi, Yuewen Wang, Xu Chao

**Affiliations:** ^1^ Shaanxi University of Chinese Medicine, Xianyang, China; ^2^ The Second Affiliated Hospital of Shaanxi University of Traditional Chinese Medicine, Xianyang, China

**Keywords:** traditional Chinese medicine, bolbostemma paniculatum, tu bei mu, hepatocellular carcinoma, network pharmacology

In the published article, there was an error in the **Funding** statement. We missed to include the funding support from the Education Department of Shaanxi Provincial Government of China (20JK0588). The correct Funding statement appears below.

“This study was supported by the fund from the Subject Innovation Team of Shaanxi University of Chinese Medicine (2019YS05), the Basic Scientific Research Project of Shaanxi Province (2020JQ-867) and the Education Department of Shaanxi Provincial Government of China (20JK0588)”.

The authors apologize for this error and state that this does not change the scientific conclusions of the article in any way. The original article has been updated.

